# Plasma neurofilament light chain and glial fibrillary acidic protein predict stroke in CADASIL

**DOI:** 10.1186/s12974-020-01813-5

**Published:** 2020-04-22

**Authors:** Chih-Hao Chen, Yu-Wen Cheng, Ya-Fang Chen, Sung-Chun Tang, Jiann-Shing Jeng

**Affiliations:** 1 Stroke Center and Department of Neurology, National Taiwan University Hospital, No. 7, Chung-Shan South Road, Taipei, 10055 Taiwan; 2 Graduate Institute of Epidemiology and Preventive Medicine (CHC), College of Public Health, National Taiwan University, Taipei, Taiwan; 3 Department of Neurology, National Taiwan University Hospital Hsinchu Branch, Hsinchu, Taiwan; 4 Department of Medical Imaging (YFC), National Taiwan University Hospital, Taipei, Taiwan

**Keywords:** CADASIL, Stroke, Intracerebral hemorrhage, Biomarkers, Neurofilament light chain, Glial fibrillary acidic protein

## Abstract

**Background:**

Stroke remains the most cumbersome disease burden in patients with cerebral autosomal dominant arteriopathy with subcortical infarcts and leukoencephalopathy (CADASIL). This study aimed to investigate whether plasma biomarkers can reflect disease severity and predict stroke recurrence in CADASIL patients.

**Methods:**

Sixty-three CADASIL patients (mean age 58.9 ± 9.3 years old, male 63%) from a multicenter registry and 17 controls were recruited. Plasma biomarkers, namely neurofilament light chain (NfL), glial fibrillary acidic protein (GFAP), tau, and ubiquitin carboxy-terminal hydrolase L1 (UCHL1), were measured using an ultra-sensitive single molecule array at baseline. Neuroimaging markers assessed included the Fazekas scale of white matter hyperintensity, numbers of lacunes, and cerebral microbleeds (CMBs). Cox proportional hazards regression models were applied to calculate the hazard ratio (HR) of plasma biomarkers at baseline for predicting incident stroke during follow-up.

**Results:**

Plasma NfL, GFAP, and UCHL1 levels were significantly elevated in the CADASIL patients than in the controls. Among the CADASIL patients, both plasma NfL and GFAP levels positively correlated with the numbers of CMBs (*r* = 0.32 and *r* = 0.37, respectively; both *p* < 0.05). Higher plasma levels of NfL and GFAP were associated with any stroke (odds ratio 2.02, 95% confidence interval [CI] 1.06–3.87) and ICH (odds ratio 2.06, 95% CI 1.26–3.35) at baseline, respectively. Within a mean follow-up period of 3.1 ± 2.1 years, 10 patients (16%) had incident stroke and 6 of them were ICH. Higher baseline NfL (HR 1.93, 95% CI 1.19–3.13) predicted any incident stroke, whereas higher GFAP (HR 2.80, 95% CI 1.21–6.53) predicted incident ICH.

**Conclusions:**

In CADASIL patients, plasma NfL can be a promising biomarker for monitoring incident stroke, whereas GFAP may have a role in cerebral hemorrhage.

## Background

Cerebral autosomal dominant arteriopathy with subcortical infarcts and leukoencephalopathy (CADASIL) is the most common hereditary cerebral small vessel disease caused by mutations in the *NOTCH3* gene, leading to devastating disease burden with stroke and vascular dementia in the affected adults [1]. Neuroimaging features such as white matter hyperintensity (WMH), lacunes, and cerebral microbleeds (CMBs) may occur 10 to 15 years before the onset of stroke or cognitive decline [1]. In East Asian, p.R544C in exon 11 on *NOTCH3* gene is the most prevalent hot spot mutation and accounted for more than 70% of the patients in their CADASIL cohorts [2, 3]. Regarding the vascular events, ischemic stroke (IS) or transient ischemic attack is considered the cardinal features in CADASIL. Despite that intracerebral hemorrhage (ICH) is considered a rare manifestation in Caucasian CADASIL patients, a significant proportion of East Asian patients harboring p.R544C *NOTCH3* mutation also suffer from ICH, and those with ICH are more prone to have recurrent stroke [4, 5]. Although the natural course of the disease has been extensively depicted, establishing reliable biomarkers to predict the occurrence of a vascular event including IS and ICH is crucial for developing an effective prevention strategy.

Studies have suggested that the brain parenchymal fraction or lacunes best correlate with disease severity and predict clinical worsening in CADASIL [6–9]. Fluid biomarkers, especially blood-based, have the advantage of easy collection and repeat measurement over the relatively inconvenient neuroimaging tests. In CADASIL patients, the neurofilament light chain (NfL) blood level has been found to correlate with the clinical and neuroimaging burdens [10] and can predict their long-term disability and survival [11]. Fluid biomarkers are used for predicting the occurrences of IS and ICH in CADASIL patients; however, they have not been well studied. Glial fibrillary acidic protein (GFAP), a brain-specific intermediate filament protein produced by astrocytes in response to brain injury, has been found to be a reliable marker for differentiating ICH and IS in patients with acute stroke [12, 13]. Ubiquitin carboxyl-terminal hydrolase L1 (UCHL1), a neuronal cytoplasmic deubiquitinating enzyme, is reported to be elevated in acute IS [14, 15]. Tau protein is a well-known marker of Alzheimer disease (AD) but is also a potential biomarker of acute stroke [16, 17].

Recently, a panel of ultra-sensitive immunoassays targeting the aforementioned blood-based biomarkers has been developed [18–21]. It has the advantages of assessing multiple biomarkers at once and comparing their performance based on the clinical interests. We aimed to use this panel to investigate whether these blood-based biomarkers can reflect disease severity, correctly identify stroke event, and predict its incidence in CADASIL patients.

## Methods

### Standard protocol approval, registrations, and patient consents

This study was approved by the ethics committees of all the participating hospitals on the understanding that all data would be coded, and patient anonymity would be guaranteed. Written informed consent was obtained from all patients and/or their relatives.

### Participants and clinical information

This study recruited 68 CADASIL patients from 8 hospitals in Taiwan. Patients were screened for *NOTCH3* mutation if they had clinical and neuroimaging evidences suggestive of cerebral small vessel disease [22]. The initial manifestations or reasons for screening for *NOTCH3* mutation in these patients were as follows: stroke in 52 patients, cognitive or gait deterioration in 7, headache or dizziness in 5, asymptomatic family members of known CADASIL patients in 3, and incidentally found marked leukoaraiosis in 1. The patients’ blood collected from different hospitals underwent genetic diagnosed centrally by combining p.R544C hot-spot mutation screening followed by sequencing of the most frequently mutated *NOTCH3 exons* (3, 4, 5, 6, 11, 18) if p.R544C was not detected. Sixty-five patients (96%) had *NOTCH3* mutation on exon 11 p.R544C. Demographic data of the CADASIL patients, including age, gender, smoking history, and medical history of hypertension, diabetes mellitus, dyslipidemia, and headache, were recorded. Family history of stroke was defined as a patient having any first-degree relative who had experienced stroke. In addition, 17 participants without neurological symptoms and signs were recruited from the cardiovascular department of the same hospitals as the control group.

Stroke was defined as an acute episode of focal neurological dysfunction lasting longer than 24 h with neuroimaging revealing focal infarction or hemorrhage in the brain relevant to the symptoms. IS was defined as the observation of a new hypodensity lesion or high diffusion-weighted imaging signal intensities through computed tomography (CT) or magnetic resonance imaging (MRI), respectively, that corresponded to the clinical presentations. Asymptomatic events with only neuroimaging evidence of small or microinfarcts were not included. ICH was defined as the sudden onset of focal neurological deficit with non-traumatic intraparenchymal hemorrhage observed on head CT or MRI scans. According to our previous study, CADASIL patients with ICH as their initial onset event tended to have poor outcome [4]; therefore, we divided the CADASIL patients into three groups based on stroke history upon recruitment: those with no stroke, with IS only (IS group), and ever ICH (ICH group). All CADASIL patients received regular follow-up in the participating hospitals since they were enrolled, and any recurrent stroke events were documented.

### Measurement of plasma biomarkers

At enrollment, 10 mL venous blood was collected from each CADASIL patients and the controls. Blood samples were centrifuged (2500×*g* for 15 min) within 1 h of collection, and the plasma aliquots were stored in cryotubes at – 80 °C until testing was performed. The blood samples were analyzed through the Neurology 4-Plex assay established by the Simoa platform (Quanterix; Lexington, MA, USA). The assay could quantify the plasma concentrations of NfL, GFAP, tau, and UCHL1 simultaneously, and the measurement details are as described previously [18, 19]. Measurements were performed by board-certified laboratory technicians who were blinded to the clinical groups.

All samples were run in duplicates, and the average concentrations were calculated. In addition, two internal quality control samples were run in the beginning and at the end of each run, and the quality controls for all four biomarkers were passed. The coefficients of variations for NfL, GFAP, tau, and UCHL1 were 5.9%, 5.3%, 16.4%, and 25.7%, respectively, which are consistent with those in a previous study [19]. The lower limits of detection of NfL, GFAP, tau, and UCHL1 assays were 0.039, 0.0265, 0.011, and 3.991 pg/mL, respectively, whereas the lower levels of quantification were 0.241, 0.467, 0.053, and 5.45 pg/mL, respectively. Only 63 of 68 CADASIL patients were included in this study because the plasma volume of 1 patient sample was too low for the assay, and the remaining 4 samples had considerable interference during the assay due to extremely high fluorescence.

### Neuroimaging assessment

All patients underwent at least one CT or MRI scan of the brain. The MRI protocols included a T1- and T2-weighted image, fluid-attenuated inversion recovery (FLAIR), susceptible weighted image (SWI), and time-of-flight MR angiography. WMH was defined as abnormally high signals on T2 or FLAIR sequences. WMH was semi-quantified by using the Fazekas scale, which ranged from 0 to 3, where a higher score indicated more severe WMH change [23]. Lacune was defined as a round or ovoid, cerebrospinal fluid-filled cavity with a diameter of approximately 3−15 mm, which had increased signals on T2 or FLAIR sequences and reduced signal on T1 imaging [24]. The numbers of lacunes in each patient were counted. CMBs were defined as areas of homogeneous round signal loss and size less than 10 mm in diameter on SWI. The presence, distribution, and number of CMBs were documented using the Microbleed Anatomical Rating Scale [25]. The location of CMBs was classified as none, deep, lobar, or mixed (both deep and lobar).

Six of the 63 patients received only head CT scan. The remaining 57 patients received MRI scans that were evaluated by two readers independently (C.H.C. and S.C.T.), and a consensus decision was reached in case of disagreement between them. The intraclass correlation was 0.94 (95% confidence intervals [CI], 0.87–0.97) for the numbers of lacunes and was 0.98 (95% CI, 0.94–0.99) for the numbers of CMBs.

### Statistical analyses

Continuous variables were presented as means and standard deviations, and categorical variables were presented as numbers and percentages. Because of the right-skewed distributions, the concentrations of all four plasma biomarkers (NfL, GFAP, tau, and UCHL1) were natural log (ln) transformed before further analysis. First, the plasma levels of NfL, GFAP, tau, and UCHL1 in the CADASIL patients and the control group were compared using the Mann-Whitney *U* test. Further, the baseline characteristics and plasma levels of biomarkers in all the 63 CADASIL patients were compared with patients of no stroke, IS only, and ICH groups by using the Kruskal–Wallis test and chi-squared test as appropriate.

To determine whether plasma biomarkers were associated with clinical outcome, multivariable logistic regression analyses were performed to estimate the adjusted odds ratio (aOR) and the 95% CI, and age, sex, and hypertension were forced as covariates. Three models were applied, in which the independent variables were plasma biomarkers, while the dependent variables included a diagnosis of CADASIL (versus control), having stroke at baseline (versus no stroke), and having ICH at baseline (versus no ICH), respectively.

Next, the Spearman rank sum test was applied to explore nonparametric correlations between plasma biomarkers and the severity of neuroimaging feature. Multiple linear regression models were used to assess the effects of plasma biomarkers on the neuroimaging features, with age, sex, and hypertension as covariates. In the models, the number of lacunes was square root transformed, and the number of CMBs was log_10_ transformed to obtain normal distributions.

Finally, the Cox proportional hazards regression models were applied to calculate the hazard ratio (HR) of plasma biomarkers at baseline for predicting any incident stroke and ICH during follow-up. Because our previous research revealed that the baseline ICH was predictive of recurrent stroke, we adjusted the baseline ICH in the Cox models in addition to age and sex. In addition, receiver operating characteristic (ROC) curve was plotted to explore the ability of individual biomarkers in predicting incident stroke and ICH, and estimates of area under curve (AUC) were obtained. Kaplan–Meier curves of incident stroke- or incident ICH-free survival during the follow-up period were plotted between the CADASIL patients with levels of plasma biomarkers above and below the upper tertile, and the log rank test was used to determine statistical differences between the groups. A *p* value of < 0.05 indicated statistical significance. All statistical analyses were performed using SAS version 9.4 (SAS Institute Inc, Cary, NC, USA).

## Results

### Baseline characteristics of the study participants

The current study analyzed 63 CADASIL patients with genetically confirmed *NOTCH3* mutation. The average age of the patients at inclusion was 58.9 ± 9.3 years, and 40 (63.5%) patients were men. Sixteen (25.4%) patients did not have clinically documented stroke events at baseline (no stroke group), 26 (41.3%) had only IS (IS group), and 21 (33.3%) had at least one symptomatic hemorrhagic stroke (ICH group) at baseline. Hypertension was more prevalent in patients with stroke, especially in the ICH group (*p* = 0.001). Other clinical history was not significantly different among the three groups. Of the neuroimaging features, the ICH group had more severe WMH, higher numbers of CMBs, and more mixed location of CMBs (Table 1).

**Table 1:** Comparison between different groups of CADASIL patients

	No stroke (*n* = 16)	IS only (*n* = 26)	Ever ICH (*n* = 21)	*p* value
Age (years)	56.1 ± 9.2	59.0 ± 9.4	60.7 ± 9.3	0.22
Sex	11 (68.8%)	15 (57.7%)	14 (66.7%)	0.72
Hypertension	3 (18.8%)	14 (53.9%)	17 (81.0%)	0.001
Diabetes mellitus	3 (18.8%)	9 (34.6%)	3 (14.3%)	0.25
Hyperlipidemia	7 (43.8%)	9 (34.6%)	9 (42.9%)	0.79
Ever smoking	3 (18.8%)	8 (30.8%)	8 (38.1%)	0.47
Headache	4 (25.0%)	2 (15.4%)	1 (7.1%)	0.45
Plasma biomarkers				
NfL	2.53 ± 0.90	3.49 ± 1.38	3.46 ±1.24	0.03
GFAP	4.42 ± 0.40	4.95 ± 1.31	5.89 ± 1.66	0.01
Tau	− 0.74 ± 1.17	− 0.21 ± 1.11	− 0.15 ± 1.34	0.29
UCHL1	2.16 ± 0.73	2.40 ± 1.24	3.07 ± 1.66	0.07
Neuroimaging markers				
WMH*	2.2 ± 1.0	2.0 ± 0.9	2.8 ± 0.3	0.04
Number of lacunes*	3.8 ± 4.3	6.2 ± 5.4	4.7 ± 4.6	0.30
Number of CMBs^†^	15.0 ± 32.2	14.5 ± 19.7	32.2 ± 29.3	0.02
Location of CMBs none/lobar/deep/mixed	5/1/2/6	5/0/7/9	0/0/1/12	0.02
Follow-up				
Total duration (years)	2.11 ± 0.31	3.20 ± 1.62	3.78 ± 2.73	0.09
Recurrent stroke	0 (0%)	3 (11.5%)	7 (33.3%)	0.02

Data are expressed in mean ± standard deviation or number (percentage)

*CADASIL* cerebral autosomal dominant arteriopathy with subcortical infarcts and leukoencephalopathy, *CMB* cerebral microbleed, *GFAP* glial fibrillary acidic protein, *ICH* intracerebral hemorrhage, *IS* ischemic stroke, *NfL* neurofilament light chain, *UCHL1* ubiquitin carboxy-terminal hydrolase L1, *WMH* white matter hyperintensity

*n = 16, 25, and 16 for each group; ^†^*n* = 14, 21, and 13 for each group

**Fig. 1:**
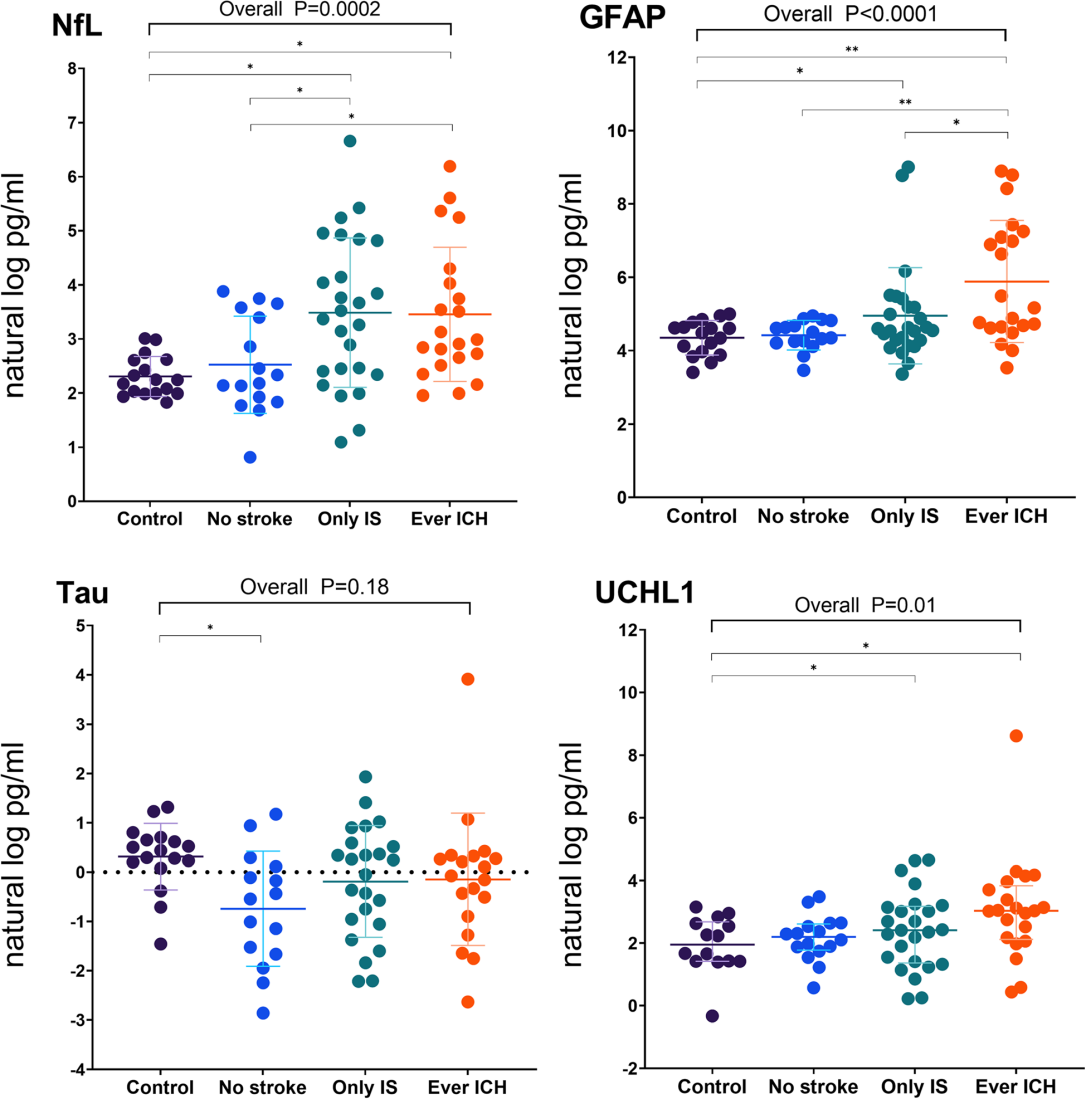
Plasma biomarker levels in the CADASIL and control groups. NfL, GFAP, and UCHL1 levels were higher in the patients than in the control group. NfL was higher in the patients with stroke (IS only or ever ICH). GFAP was the highest in the patients with ICH. *p < 0.05, **p < 0.01

The natural log-transformed plasma biomarkers levels of the control and each stroke subgroup of the CADASIL patients are plotted in Fig. 1. The age-adjusted mean plasma levels of the biomarkers were generally higher in the CADASIL patients than in the control group (NfL 3.30 ±0.28 vs 2.08 ± 0.56 natural log pg/mL; GFAP 5.21 ± 0.30 vs 4.04 ± 0.60 natural log pg/mL; UCHL1 2.64 ± 0.30 vs 1.61 ± 0.65 natural log pg/mL; all *p* < 0.01), except for those of tau (− 0.30 ± 0.29 vs 0.22 ± 0.56 natural log pg/mL, *p* = 0.11).

### Relationship between plasma biomarkers and stroke subtype

In patients with history of stroke, the time intervals between the latest stroke and blood sampling were 415.5 ± 899.8 days. A non-significant trend of negative correlation between the intervals and levels of NfL (Spearman *ρ* = − 0.25, 95% CI = − 0.51–0.05) and GFAP (*ρ* = − 0.10, 95% CI = − 0.39–0.20) was observed. Among CADASIL patients, the plasma NfL level was higher in the IS (3.53 ± 0.42 natural log pg/mL) and ICH (3.44 ± 0.47 natural log pg/mL) groups than in the no stroke group (2.68 ± 0.56 natural log pg/mL, *p* = 0.01 and *p* = 0.04, respectively), whereas the GFAP level was elevated in the ICH group (5.87 ± 0.49 natural log pg/mL) than in the IS (5.01 ± 0.44 natural log pg/mL, *p* = 0.01) or no stroke group (4.62 ± 0.57 natural log pg/mL, *p* = 0.001).

The multivariable logistic regression analysis differentiated the CADASIL patients from the control group based on higher plasma levels of NfL (aOR 12.4, 95% CI 2.87–53.1, *p* = 0.001), GFAP (aOR 27.9, 95% CI 2.94–265.4, *p* = 0.004), and UCHL1 (aOR 4.08, 95% CI 1.57–10.6, *p* = 0.004). We investigated which plasma biomarkers were associated with the history of stroke (*n* = 47) and ICH (*n* = 21) at baseline in the CADASIL patients. After adjusting for age, sex, and hypertension, the higher NfL level was found to be associated with any stroke at baseline (aOR 2.02, 95% CI 1.06–3.87, *p* = 0.03), whereas the GFAP level had a borderline association (aOR 2.51, 95% CI 0.92–6.89, *p* = 0.07). By contrast, only the plasma GFAP level was found to be associated with ICH at baseline (aOR 2.06, 95% CI 1.26–3.35, *p* = 0.004; Table 2).

**Table 2:** Plasma biomarkers predicting stroke and ICH

	Stroke at baseline		ICH at baseline		Incident stroke		Incident ICH	
	aOR (95% CI)*	*p* value	aOR (95% CI)*	*p* value	HR (95% CI)^†^	*p* value	HR (95% CI)^†^	*p* value
NfL	**2.02 (1.06**–**3.87)**	**0.03**	1.17 (0.76–1.81)	0.48	**1.93 (1.19**–**3.13)**	**0.01**	**2.39 (1.21**–**4.70)**	**0.01**
GFAP	2.51 (0.92–6.89)	0.07	**2.06 (1.26**–**3.35)**	**0.004**	1.62 (0.92–2.85)	0.09	**2.80 (1.21**–**6.53)**	**0.02**
Tau	1.52 (0.85–2.71)	0.15	1.23 (0.76–1.99)	0.40	0.74 (0.37–1.47)	0.39	1.36 (0.61–3.04)	0.45
UCHL1	1.28 (0.72–2.28)	0.39	1.74 (0.995–3.06)	0.05	1.42 (0.80–2.52)	0.23	1.61 (0.86–3.02)	0.14

Numbers in bold indicate statistical significance

*aOR* adjusted odds ratio, *GFAP* glial fibrillary acidic protein, *HR* hazard ratio, *ICH* intracerebral hemorrhage, *NfL* neurofilament light chain, *UCHL1* ubiquitin carboxy-terminal hydrolase L1

*Logistic regression models adjusted for age, sex, and hypertension. ^†^Cox regression models adjusted after age, sex, and history of intracerebral hemorrhage at baseline

### Relationship between plasma biomarkers and neuroimaging features

Then time intervals between blood sampling and neuroimaging were 70.9 ± 94.6 days. The number of CMBs was positively correlated with the plasma levels of both NfL (*ρ* = 0.32, 95% CI 0.03–0.56, *p* = 0.03) and GFAP (*ρ* = 0.37, 95% CI = 0.08–0.60, *p* = 0.01). In the multiple linear regression analysis, similar finding was observed that higher plasma NfL was associated with a higher number of CMBs (*β* = 0.16, 95% CI 0.02–0.30, *p* = 0.02). No significant associations were observed between plasma biomarkers and other neuroimaging markers such as severity of WMH and numbers of lacunes (Table 3).

**Table 3:** Associations between plasma biomarkers and neuroimaging indicators

		Fazekas score (*n* = 57)	Lacunes (*n* = 57)	CMBs (*n* = 47)
NfL	*ρ* (95% CI)*	0.02 (− 0.26, 0.29)	0.09 (− 0.19, 0.36)	**0.32 (0.03, 0.56)**
	*β* (95% CI)^†^	0.03 (− 0.14, 0.19)	0.09 (− 0.17, 0.35)	**0.16 (0.02, 0.30)**
GFAP	*ρ* (95% CI)*	0.23 (− 0.05, 0.47)	− 0.07 (− 0.34, 0.21)	**0.37 (0.08, 0.60)**
	*β* (95% CI)^†^	0.05 (− 0.13, 0.23)	− 0.03 (− 0.31, 0.25)	0.15 (− 0.02, 0.33)
Tau	*ρ* (95% CI)*	− 0.08 (− 0.35, 0.20)	0.08 (− 0.20, 0.34)	0.08 (− 0.23, 0.36)
	*β* (95% CI)^†^	− 0.01 (− 0.19, 0.18)	0.07 (− 0.21, 0.35)	0.08 (− 0.08, 0.24)
UCHL1	*ρ* (95% CI)*	0.01 (− 0.27, 0.29)	− 0.13 (− 0.39, 0.16)	0.08 (− 0.23, 0.36)
	*β* (95% CI)^†^	0.02 (− 0.15, 0.20)	− 0.13 (− 0.41, 0.14)	0.06 (− 0.10, 0.21)

Numbers in bold indicate statistical significance

*CMBs* cerebral microbleeds, *GFAP* glial fibrillary acidic protein, *NfL* neurofilament light chain, *UCHL1* ubiquitin carboxy-terminal hydrolase L1

*Spearman’s rank correlation partially adjusted for age and sex. ^†^Multiple linear regression analysis adjusted for age, sex, and hypertension

### Impacts of plasma biomarkers on incident stroke and ICH

**Fig. 2:**
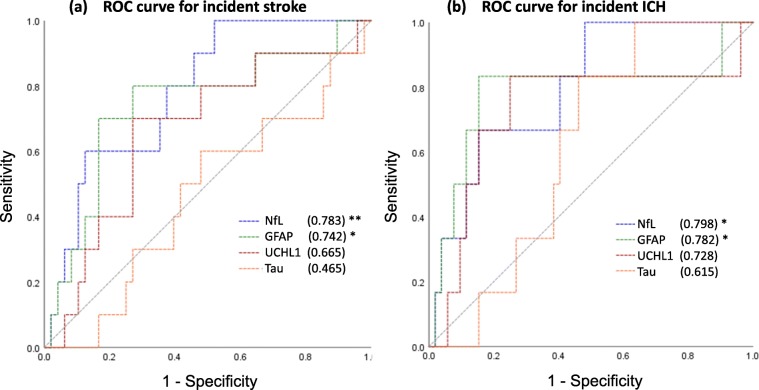
Receiver operating characteristics curves in predicting **a** incident stroke and **b** incident ICH. The area under curve (AUC) was calculated and presented in the parenthesis and was test against the default level of 0.5 (indicating no discrimination). *p < 0.05, **p < 0.01

During a mean follow-up period of 3.1 ± 2.1 years, 10 patients (16%) had at least 1 incident stroke, and 6 of them were ICH. The annual stroke and ICH rates were 8.1 and 4.8 per 100 person-years, respectively. In Cox regression analysis after adjusting for age, sex, and hypertension, NfL predicted the incident stroke (HR 1.93 per 1-unit increase in natural log-transformed NfL, 95% CI 1.19–3.13, *p* = 0.01). The proportional hazards assumption was not violated. In addition, both higher NfL (HR 2.39, 95% CI 1.21–4.70, *p* = 0.01) and GFAP (HR 2.80, 95% CI 1.21–6.53, *p* = 0.02; Table 2) levels were predictive of incident ICH during follow-up. The results remained consistent when the plasma biomarkers were analyzed in their original scale (HR 1.004 per 1-unit increase in the absolute NfL level, 95% CI 1.000–1.008, *p* = 0.03; HR 1.001 per 1-unit increase in the absolute GFAP level, 95% CI 1.000–1.002, *p* = 0.048). Other plasma biomarkers did not predict recurrent stroke. When plotting the ROC curve, plasma NfL and GFAP remained the only two plasma biomarkers that had ability in predicting incident stroke (AUC = 0.783 and 0.742 for NfL and GFAP, respectively, both *p* < 0.05) or ICH (AUC = 0.798 and 0.782 for NfL and GFAP, respectively, both *p* < 0.05) (Fig. 2).

**Fig. 3:**
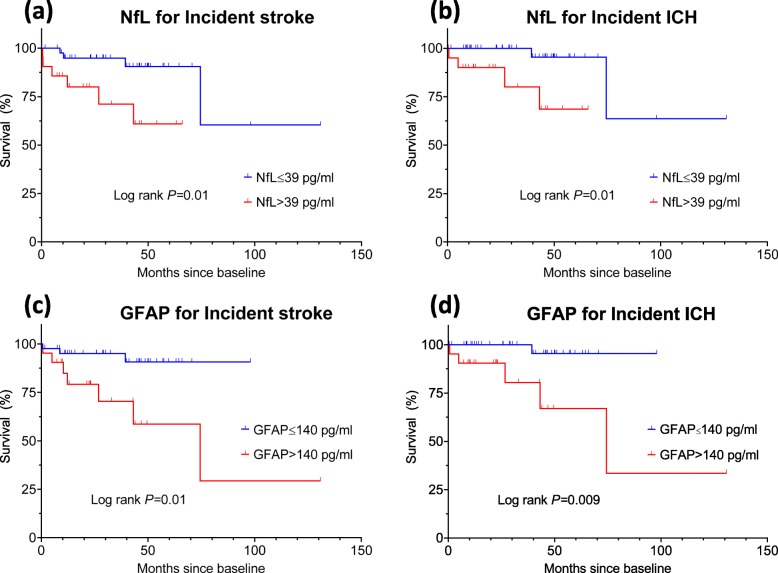
Kaplan–Meier plots of incident stroke or ICH-free survival between patients with baseline biomarkers of the highest tertile (red line) or below (blue line). **a** NfL and incident stroke. **b** NfL and incident ICH. **c** GFAP and incident stroke. **d** GFAP and incident ICH

In the Kaplan–Meier plot (Fig. 3), patients with the highest NfL tertile (> 39 pg/mL) were found to be associated with higher risks of incident stroke (log rank *p* = 0.01) and ICH (*p* = 0.01). Similarly, patients with the highest GFAP tertile (> 140 pg/mL) were at a risk of both incident stroke (*p* = 0.01) and ICH (*p* = 0.009).

## Discussion

The highlight of the present study was that it demonstrated that plasma biomarkers, NfL and GFAP, had the ability to identify stroke or ICH events in CADASIL patients. At the baseline, the plasma NfL and GFAP levels were elevated in the IS and ever ICH groups, respectively. The plasma NfL and GFAP levels were also positively associated with the numbers of CMBs. Moreover, in a prospective follow-up, higher NfL was predictive of incident stroke, and GFAP mainly predicted cerebral hemorrhage. The plasma UCHL1 and tau levels did not contribute significantly in the current analysis.

Stroke is the most cumbersome disease burden in CADASIL patients. More than 50% of CADASIL patients had stroke or transient ischemic attack as initial manifestations, and 70% may have ischemic events in their lifetime [26, 27]. Studies on CADASIL biomarkers have usually focused on their association with neuropsychological performance, functional status, or neuroimaging features [6, 7, 28]. The number of CMBs had been reported to predict incident IS [29]. However, studies on the influence of fluid biomarkers on stroke events are scarce.

NfL, a major component of the neuronal cytoskeleton in the axons, might be released into the cerebrospinal fluid and blood upon tissue damage in the central nervous system. In our cohort, the NfL level was elevated in both IS and hemorrhagic stroke patients and was also modestly correlated with the CMBs load. These findings indicated that NfL levels reflect structural axonal damage in the brains, irrespectively of the underlying insults. The application of the blood-based biomarker NfL has gained considerable attention not only in the research field of neurodegenerative disorders such as multiple sclerosis [30], AD [31], or Parkinsonism disorders [32] but also in acute IS [33]. Because CADASIL is a neurodegenerative disease intermixed with acute stroke events, NfL can be a promising biomarker for monitoring its disease burden. One study found that in CADASIL patients, the serum NfL level was highly associated with the brain MRI markers such as WMH, CMBs, or mean diffusivity and had independent effects on processing speed, severity of focal neurological deficits, and functional disability [10]. Another study revealed that the serum NfL level not only correlated with cognition and functional disabilities at baseline but also predicted their longitudinal progression and overall survival [11]. Our study extended upon the previous findings by demonstrating that NfL can also be a useful marker for predicting stroke event in the future. Although neuroimaging lesion load or cognitive function was crucial prognostic factors in CADASIL, overt stroke event might engender greater threat to both patients and clinicians. Determining the blood NfL level can offer practical clinical information for identifying patients at high risk.

Another novel finding of this study was that the plasma GFAP level can be a crucial biomarker for identifying and predicting the ICH event. ICH was infrequently reported in CADASIL patients of the Western countries, whereas in the East Asian patients, especially those with p.R544C mutation of *NOTCH3*, a risk of lifetime ICH was noted to be as high as 40% [3, 4, 34]. In addition to the distribution or number of CMBs [4, 34], no other biomarkers have been found to be associated with CADASIL-related ICH. GFAP, a glial-specific biomarker that is known to be preferentially elevated during hemorrhagic brain insult, including spontaneous ICH or traumatic brain injury [13, 35, 36], could differentiate ICH from IS even in the early phase. Similarly, we found that the GFAP level was notably elevated in ICH patients and correlated with the numbers of CMBs. We also demonstrated that a higher GFAP level was principally associated with baseline and subsequent hemorrhagic stroke in CADASIL patients. In the post-mortem brain of CADASIL patients, increased numbers of GFAP-positive astrocytes co-localized with autophagy markers in the WMH region, indicating that astrocytopathy might contribute to the severity of the small vessel disease [37]. Because the CADASIL patients with ICH tended to have more severe clinical and neuroimaging phenotypes, a higher baseline plasma GFAP level could be a surrogate marker, reflecting the disease burden and bleeding risk in the future.

Although UCHL1 did not provide useful prognostic information in our study, its level was elevated in the CADASIL patients with stroke, especially the ICH group. The higher UCHL1 level was found to be marginally associated with ICH (aOR 1.74, 95% CI 0.995–3.06, *p* = 0.05). This is in concordance with previous study that the UCHL1 level was higher in ICH patients than in the IS or control group [14]. Unlike GFAP, UCHL1, a neuronal-specific enzyme involved in the ubiquitin-proteasome pathway, has a significant role in the brain’s self-repair mechanisms after injury [38, 39]. However, the associations between the UCHL1 level and clinical or neuroimaging severity were not significant. Further study with a larger sample size might provide considerable insights into the influence of UCHL1 on CADASIL patients. By contrast, the plasma levels of tau were not different between the control and patient groups, and thus were not useful in such a clinical setting.

The current study enrolled CADASIL patients from Taiwan where p.R544C on exon 11 mutation of the *NOTCH3* gene was most prevalent. Registry-based studies showed that around 2.1 to 2.8% of stroke patients in Taiwan may harbor p.R544C *NOTCH3* mutation and harboring the p.R544C resulted in a three-fold increased risk for stroke [5, 40]. Besides, the prevalence of p.R544C mutation was estimated at 0.9% in general population in Taiwan [40]. It may explain why that p.R544C mutation accounted for more than 90% of the enrolled patients in the current study. The clustering of mutations in the sampled patients offered advantages in controlling otherwise unknown genotype-phenotype heterogeneity in outcome; nevertheless, it may limit the generalizability to CADASIL patients with other mutation points in whom ICH occurred rarely.

The strengths of our study included the application of a composite panel of relevant blood biomarkers at once. We not only assessed cross-section associations between plasma biomarkers and clinical or neuroimaging features but also set up a prospective follow-up to detect any meaningful stroke event. Applying a multiplex panel comprised of different biomarkers offered the advantages in detecting relevant targets and satisfying clinical needs simultaneously and efficiently. The multiplex panel of neurodegenerative biomarkers had been applied increasingly in clinical setting such as post-cardiac surgery, traumatic brain injury, sport-related concussion, or frontotemporal dementia [18–21]. To our knowledge, this was the first application of the multiplex panel in CADASIL. Further validation of our findings was warranted to see whether NfL and GFAP could serve as biomarkers in identifying patients at risk of stroke.

However, several other limitations still existed. First, a relatively small sample size might have weakened its statistical power and limited further subgroup analysis. Second, not all clinical and neuroimaging features were added into the regression models. This was because not all patients had received MRI scans, and the relatively few outcomes did not permit several variables in the models. We added all relevant neuroimaging variables and applied a stepwise selection method, and the results remained consistent, that is, NfL was associated with stroke (OR 2.12, 95% CI 1.08–4.18), and GFAP was associated with ICH (OR 1.87, 95% CI 1.00–3.52). Third, we have tested different outcome variables simultaneously without adjustment of multiple testing. We acknowledged this limitation, while considered current study as a rather exploratory analysis and did not set prespecified restriction on multiple testing. Fourth, as above, our results may not be generalizable to other ethnicity because certain genotype–phenotype correlations contributed to the higher incidence of ICH in our CADASIL cohort. Finally, we only measured the plasma biomarker levels at baseline. Further study encompassing a repeat measurement of biomarker levels might provide a greater insight into their influence.

## Conclusion

In conclusion, we demonstrated the feasibility of using blood-based biomarkers for identifying and predicting stroke events in CADASIL patients. The advantage of single-time measurement of the levels of plasma biomarkers, such as NfL and GFAP, is that it can be used to monitor disease severity and predict clinical events. Further study with a larger sample size is required to confirm the association of the plasma biomarkers with both IS and ICH in CADASIL patients.

## Data Availability

The datasets used and/or analyzed during the current study are available from the corresponding author on reasonable request.

## Abbreviations

AUC: Area under curve
CADASIL: Cerebral autosomal dominant arteriopathy with subcortical infarcts and leukoencephalopathy
CMBs: Cerebral microbleeds
CT: Computed tomography
FLAIR: Fluid-attenuated inversion recovery
GFAP: Glial fibrillary acidic protein
IS: Ischemic stroke
ICH: Intracerebral hemorrhage
MRI: Magnetic resonance imaging
NfL: Neurofilament light chain
ROC: Receiver operating characteristics
UCHL1: Ubiquitin carboxy-terminal hydrolase L1
WMH: White matter hyperintensity
